# Smoking Initiation Among Young Adults in the United States and Canada, 1998-2010: A Systematic Review

**Published:** 2011-12-15

**Authors:** Kit S. Freedman, Nanette M. Nelson, Laura L. Feldman

**Affiliations:** Wyoming Survey & Analysis Center at the University of Wyoming; Wyoming Survey & Analysis Center at the University of Wyoming, Laramie, Wyoming; Wyoming Survey & Analysis Center at the University of Wyoming, Laramie, Wyoming

## Abstract

**Introduction:**

Young adults have the highest smoking rate of any age group in the United States and Canada, and recent data indicate that they often initiate smoking as young adults. The objective of this study was to systematically review peer-reviewed articles on cigarette smoking initiation and effective prevention efforts among young adults.

**Methods:**

We searched 5 databases for research articles published in English between 1998 and 2010 on smoking initiation among young adults (aged 18-25) living in the United States or Canada. We extracted the following data from each study selected: the measure of initiation used, age range of initiation, age range of study population, data source, target population, sampling method, and sample size. We summarized the primary findings of each study according to 3 research questions and categories of data (eg, sociodemographic) that emerged during the data extraction process.

**Results:**

Of 1,072 identified studies, we found 27 articles that met our search criteria, but several included a larger age range of initiation (eg, 18-30, 18-36) than we initially intended to include. Disparities in young adult smoking initiation existed according to sex, race, and educational attainment. The use of alcohol and illegal drugs was associated with smoking initiation. The risk of smoking initiation among young adults increased under the following circumstances: exposure to smoking, boredom or stress while serving in the military, attending tobacco-sponsored social events while in college, and exposure to social norms and perceptions that encourage smoking. Effective prevention efforts include exposure to counter-marketing, denormalization campaigns, taxation, and the presence of smoke-free policies.

**Conclusion:**

Much remains to be learned about young adult smoking initiation, particularly among young adults in the straight-to-work population. Dissimilar measures of smoking initiation limit our knowledge about smoking initiation among young adults. We recommend developing a standardized measure of initiation that indicates progression to regular established smoking.

## Introduction

Young adulthood represents a critical time in the transition from adolescence to adulthood, when changes in risk-taking behaviors such as experimenting with smoking become apparent (1,2). According to the 2009 National Health Interview Survey (NHIS), 38% of current smokers aged 18 to 25 report they initiated regular smoking after age 18, a 27% increase from the 2007 NHIS estimate of 30% (3,4). In the 2008 National Survey on Drug Use and Health (NSDUH), 1 million people reported that they initiated smoking as young adults, an increase from about 600,000 in 2002; during the same period, the increase in the number of initiates aged 18 or younger was considerably less, from 1.3 million to 1.4 million (5).

Until 1998, when Wechsler et al (6) reported that 28% of college students who smoked initiated smoking at age 19 or older, most public health researchers believed that smoking behavior (ie, initiation) was established by age 18 (7). Results from a follow-up survey (8) confirmed the delayed age of initiation. Soon after, others (2,7,9) began challenging the long-held assumption that smoking initiation mostly occurs before age 18.

Despite the increased interest in young adult smoking behavior, we found no systematic review of published research on this topic, except a review in 2003 that identified a lack of prevention research and anti-tobacco initiatives for young adults (1). The objective of our study was to summarize the current knowledge on young adult smoking initiation and effective prevention efforts targeting this population.

## Methods

### Study design

We conducted a systematic review of peer-reviewed research articles published in English between January 1, 1998, and December 31, 2010, on cigarette smoking initiation among young adults living in the United States or Canada. The following 3 questions guided our research:

- Which young adults initiate smoking?
- Under what circumstances does initiation among young adults take place?
- What programming and policy efforts reduce young adult smoking initiation?

### Definitions of terms

Although we initially defined "young adults" as being aged between 18 and 25 years, we accepted other definitions in the research literature (eg, aged 18-24 y, 18-29 y, 18-36 y). We applied this definition to the age of initiation. Similarly, because we were unclear on a definition for "smoking initiation," we accepted other terms, particularly when authors used the term "initiation" to discuss the phenomenon of smoking onset or the progression from nonsmoker to experimental smoker or regular smoker. We considered articles that focused on the initiation of cigarette smoking only.

### Data sources

We searched 5 databases: Academic Search Premier, PsycINFO, MEDLINE, Health Source: Nursing/Academic Edition via the EBSCOhost search platform, and PubMed. We chose to search these databases because they are comprehensive and archive articles that are most closely related to our topic. We limited our search to articles published after January 1, 1998, because Wechsler et al published their article first documenting young adult smoking initiation in 1998. We used the medical subject headings (MeSH) and free search terms "smoking (and) tobacco (and) prevention" crossed with terms identifying our population of interest, "young adult," and "initiation (or) uptake (or) onset." We included variations of the original search terms (eg, "young adult smoking [and] initiation [or] uptake") to ensure our search was comprehensive. Additionally, we included the term "policy" when searching for articles related to the research question on programming and policy.

### Study selection

The initial search identified 1,072 peer-reviewed articles; of these, we identified 87 for further review. We excluded 985 articles on the basis of their title or abstract: 348 were duplicate entries, 232 focused on unrelated topics, 151 did not report on smoking initiation or on young adults, 102 were rooted in biomedical or genetic research or both, 77 studied populations outside the United States and Canada, and 75 studied other types of tobacco (eg, smokeless tobacco, cigars) or nontobacco substances (eg, marijuana, cocaine).

Next, we reviewed all remaining abstracts and obtained full-text versions for 85 articles; full-text versions were not available for 2 studies (15,16). For these 2 studies, we analyzed the abstracts, which provided sufficient information to continue with the data extraction process. Two reviewers (K.S.F., N.M.N.) independently read the articles and coded them for inclusion or exclusion. Following independent review, the 2 reviewers met to discuss preliminary findings and to reach a consensus on the studies to be included. Throughout the initial review process, we also coded some of the excluded articles as "background" because although they did not meet our exact inclusion criteria, they were otherwise relevant to young adult smoking initiation and could inform our understanding of the issue. Of the 87 articles (including 2 abstracts) reviewed, we excluded 69 for the following reasons: 12 did not focus on young adults, 23 did not focus on smoking initiation, 11 reported on findings not relevant to our research questions, and 9 focused on other types of tobacco or nontobacco. We set aside 14 articles as background. In addition, we identified 9 articles through the list of references in the reviewed articles. Thus, we had a total of 27 articles (including 2 abstracts) for further analysis.

### Data extraction

Following study selection, the same 2 reviewers jointly extracted information on study design and results from the 27 selected articles. We organized the information into the following categories: target population, measure of smoking initiation, age range of initiation, age range of sample population, data source, sampling method, and sample size. We created subcategories for measures of smoking initiation when patterns emerged. We then summarized the primary results of each study according to each of our 3 research questions and broad topical categories that emerged during the data extraction process and compiled information on significant results (*P* ≤ .05). We evaluated the generalizability of study results by considering the study design and other information. We focused on describing results that could be generalized to broader populations but noted studies of narrower scope when they provided corroborating evidence.

## Results

Overall, the 27 studies included in this review (Table 1) identified 4 target populations: young adults in the general population (n = 14), college/university students (n = 9), military personnel (n = 3), and straight-to-work (STW) young adults (ie, young adults who are not enrolled in college or enlisted in the military) (n = 1). The measure of smoking initiation varied among the studies (Table 2).

### Which young adults initiate smoking?

Young adult men are more likely than young adult women to initiate smoking as young adults (2,10-12), and nonwhite adult smokers, especially Asian/Pacific Islanders (14) and African Americans (9,13-18), are more likely to initiate as young adults than as youth (Table 3). White young adults who attend college or enlist in the military, however, are more likely to initiate or progress in their smoking behavior while in college or in the military (19-21). Additionally, young adults who report experimenting with smoking as youth (defined as "smoked at least 1 but fewer than 100 cigarettes in a lifetime and did not smoke in the last 30 days")are more likely to progress in their smoking behavior while in college (19), and young adults who report consuming alcohol (10,11,15,20,23,24,26) are also more likely to initiate smoking as young adults. We found evidence (10,11,20,26) to suggest that past illegal drug use is associated with a greater likelihood of initiating smoking among young adults in the college/university population, although this evidence cannot be generalized to the broader population.

### Under what circumstances does smoking initiation among young adults take place?

Young adults who are exposed to smoking (eg, by family members, friends, coworkers, other social contacts) are more likely to initiate smoking or to smoke than are young adults who are not exposed to smoking (27) (Table 4). Similarly, military personnel with roommates who smoke, who are bored or stressed, and who perceive their leaders or classmates to be tobacco users are more likely to initiate smoking (12,21). Furthermore, young adults in the college/university population who attend social events sponsored by the tobacco industry (28) as well as those who believe their friends approve of smoking or that experimenting with smoking is safe (19) are at increased risk of becoming smokers.

### What programming and policy efforts reduce young adult smoking initiation?

Despite a dearth of information on effective programs and policies, we found evidence that exposure to counter-industry marketing and denormalization campaigns (eg, the Truth campaign) is associated with decreased risk of smoking initiation (30,31) and less progression along a continuum of smoking intention and behavior (32) (Table 5). In addition, smoke-free policies (33) and cigarette tax increases (34) have been shown to reduce the number of young adults who initiate smoking.

## Discussion

Relative to the amount of research on young adult smoking behavior, few studies focused on the initiation of smoking among young adults. Because the 27 articles reviewed here varied widely in their research methods, ages studied, sample population, and measure of initiation, we found direct comparisons among the studies difficult to make.

Despite these challenges, this review provides information on what is known about young adults who initiate smoking and the circumstances under which they initiate. Our findings suggest that prevention efforts to reduce smoking initiation among young adults should target men and racial/ethnic minorities, especially Asian/Pacific Islanders (14) and African Americans (2,10,11,14-18), as well as college students, particularly whites (19,20,22,23), and young adults who use alcohol and other illegal drugs (10,11,15,20,23,24,26). Many studies offered guidance on the design of prevention programs and policies for the young adult population. For example, because the evidence suggests that friends influence smoking initiation among young adults, prevention programs and policies that target this population should focus on the role of peers. Because young adults with pro-smoking perceptions (eg, young adults who think that smoking is not harmful) are more likely to initiate smoking, prevention programs should also focus on education and the changing of social norms. Finally, because young adults who consume alcohol or have a history of past illegal drug use are more likely to initiate smoking as young adults, the development of comprehensive prevention programs and policies for this age group may impact an array of high-risk behaviors in addition to smoking initiation.

The lack of evidence on our question "What programming and policy efforts reduce young adult smoking initiation?" is a critical finding, given the trend in the prevention field toward evidence-based programs and outcomes. Although counter-industry marketing and denormalization strategies (30-32) are effective in preventing young adults from initiating smoking, recent evidence suggests that awareness of the national Truth campaign varies by sex and education level (35), highlighting the need for more research on the effect of these programs. Whether the lack of information on the effectiveness of prevention programs and policies indicates a general shortage of programs and policies or a lack of research, effective programs focused on reducing smoking initiation among young adults need to be identified and promoted.

The small number of articles found on military personnel and the STW population is likely explained by the easier access to college/university populations and to national survey data for the general population. Young adults in the STW group are likely diffused in the general population and not easily identified as a target group. Whatever the explanation, the limited information available on the STW population is of concern. One study found that 30% of young adults not enrolled in college (or without a college degree) smoked, compared to 14% of their college-educated counterparts (36). The study also found differences in smoking prevalence between white-collar and blue-collar workers within the STW population; those employed in the service industry and blue-collar occupations reported the highest rate of smoking prevalence among young adults aged 18 to 24, consistent with other studies (37,38). According to the US Census Bureau's 2006-2008 American Community Survey, approximately 41% of the nearly 30 million young adults aged 18 to 24 are currently enrolled in postsecondary education (39); thus, approximately 18 million young adults in the United States must be in the STW population or military (39). The STW population represents a sizeable at-risk group, about which little is known. We recommend making the young adult STW population a research priority: Who are they? Where do they work? Why do they start smoking? Answers to these questions can assist the prevention community in designing effective programs and policies.

The use of dissimilar measures for smoking initiation among studies suggests that researchers may not be obtaining an accurate picture of the prevalence of smoking initiation among the young adult population. Two studies (40,41) suggest differences in the criteria for generating prevalence rates for smoking initiation among young adults and among adolescents. The measure for adult smokers is "smoked 100 or more cigarettes in lifetime" whereas for youth and adolescents, it is "smoked 1 or more days in the past 30 days." Depending on the measure used, researchers may over- or underestimate the actual number of young adults at risk for progression to regular smoking. One study found that using the adult criteria for defining a smoker reduces the estimate of the prevalence of young adult smoking by as much as 18% (41). A growing body of evidence on the phenomenon of "occasional" or "social smokers" who may not self-identify as smokers but who, nevertheless, are at risk of becoming regular established smokers (42-46) further highlights the problems caused by dissimilar measures. Because tobacco researchers rely on measures of prevalence to identify disparities and to make policy recommendations, we need more accurate measures of prevalence for the young adult population. Moreover, because regular smoking starts with initiation, we need a measure of initiation that predicts progression to established smoking. Therefore, we recommend public health researchers work to identify a standard measure of initiation that indicates progression to established smoking.

This study had limitations. We may not have identified all relevant studies, particularly if they were not indexed in the databases used for this review. We chose to include studies only from the United States and Canada that were published in English after January 1, 1998; hence, our review does not represent research published elsewhere.

Much remains to be learned about young adult smoking initiation in the United States and Canada. The lack of information suggests that tobacco preventionists and public health researchers need to prioritize access to data on young adults, particularly those in the STW population. Moreover, dissimilar measures of smoking initiation limit what public health researchers can know about smoking initiation among young adults. Because the transition from adolescence to adulthood (ie, young adulthood) is a critical time — when changes in risk-taking behaviors become apparent — we need more research and expanded prevention efforts if we are to effect a reduction in the number of young adults who initiate smoking after age 18.

## Figures and Tables

**Table 1:** Articles Included (N = 27) in a Systematic Review of Studies on Smoking Initiation Among Young Adults in the United States and Canada, 1998-2010

**Study**	**Measure of Initiation Used in Study**	Age Initiation, y	Age of Study Population, y	**Data Source (Sample Size)**
Lantz (2)	"Age when first started to smoke fairly regularly."	Range, 19- 21	Range, 23-40	National Health Interview Survey (NHIS), 2000 (sample size not indicated)
Biener and Albers (9)	"Age when first started smoking cigarettes regularly."	Range, 18-30	Range, 18 -65	Massachusetts Adult Tobacco Survey (ATS), 1995-2000 (n = 12,449)
Reed et al (10)	"Smoked ≥100 cigarettes and smoked during the past 30 days" and "smoked <100 cigarettes but smoked during the past 30 days."	Range, 18-24	Range, 18-24	Study-specific cross-sectional survey of undergraduate students at a large urban university in southwestern United States (n = 1,113)
Myers et al (11)	"Age when first smoked a cigarette [more than a puff]?"	Range,18-23	Range, 18-23	Study-specific longitudinal study of Chinese and Korean American students at a public university in southwestern United States (n = 267)
Bray et al (12)	"Started smoking after joining the military."	Range, 18-25	Range, 18-25	Department of Defense Survey of Health Related Behaviors Among Active Duty Military Personnel, 2005 (n = 16,146)
Kiefe et al (13)	"Reported regular cigarette smoking (at least 5 cigarettes per week almost every week for at least 3 months)."	Range, 18-30	Range, 18-30	Coronary Artery Risk Development in Young Adults (CARDIA) 1985-86, 1987-88, 1990-91, 1993-94, 1995-96 (n = 3,950)
Trinidad et al (14)	"Have you smoked at least 100 cigarettes in your entire life?" and "How old were you when you first started smoking cigarettes fairly regularly?"	Range, 10-25	Range, 26-50	Tobacco Use Supplement to the Current Population Survey (TUS-CPS), 1992-93, 1995-96, 1998-99 (n = 130,356)
Viola (15)	"Smoked at least 100 cigarettes in lifetime."	Range, 18-28	Range, 18-28	NHIS, 2002 (n = 1,820)
Hailpern and Viola (16)	"Lifetime use ≥100 cigarettes."	Range, 18-24	Range, 25-34	NHIS, 1997-2003 (n = 44,137)
Trinidad et al (17)	"Have you smoked at least 100 cigarettes in your entire life?" and "How old were you when you first started smoking cigarettes fairly regularly?"	Range, 14-20	Mean, 47.9	TUS-CPS, 1992-93, 1995-96, 1998-99 (n = 512,258)
Watson et al (18)	Definition not clearly defined other than "age of smoking onset."	Mean, 18	Range, 18-39	Study-specific nonrandom, cross-sectional survey of black and white women (n = 715)
Choi et al (19)	Used a definition of smoking progression instead of initiation.^a^	Range, 18-22	Undergraduate students^c^	Teenage Attitudes and Practices Survey (TAPS) I, 1989 and TAPS II, 1993 (n = 1,479)
Reed et al (20)	"Age at which smoked first cigarette."	Range, 18-24	Range, 18-24	Study-specific cross-sectional survey of undergraduate students at a large urban university in southwestern United States (n = 1,667)
Green et al (21)	"Started smoking after joining the military."	Range, 18-36	Range, 18-36	Study-specific cross-sectional survey of United States Air Force junior-enlisted technical training students (n = 2,962)
Stockdale et al (22)	"Age when first tried cigarette smoking."	College freshmen through seniors^c^	Mean age range,19.4- 21.9	Study-specific cross-sectional surveys (2 random, 1 convenience) of students attending a university in midwestern United States (n = 1,986)
Staten et al (23)	"Age at which smoked first whole cigarette."	Range, 18-24	Range, 18-24	Study specific random, cross-sectional survey of undergraduate students at a large public university in southeastern United States (n = 437)
Tercyak et al (24)	"Ever smoked even a puff of a cigarette in the year after high school."	Mean, 18.9	Mean, 18.9	Georgetown Adolescent Tobacco Research Project and the Adult Longitudinal Outcomes and Health Assessment, 1994-2004 (n = 1,100)
Haddock et al (25)	"Smoking, even a puff, over the past 7 days."	Mean, 19.8	Mean, 19.8	Study-specific longitudinal survey of United States Air Force recruits who claimed to have never smoked cigarettes (n = 7,865)
Costa et al (26)	Never-smokers at Wave 1 (2002) but began smoking cigarettes by Wave 3 (2004).	College freshmen aged ≥18 at Wave 1 (2002)	College freshmen aged ≥18 at Wave 1 (2002)	Study-specific 3-wave longitudinal survey of undergraduate students at University of Colorado Boulder (n = 880)
Ling et al (27)	"Age when smoked first whole cigarette."	Range, 18-29	Range, 18-29	California Tobacco Survey, 2002 (n = 9,455)
Rigotti et al (28)	"Age at first use" and "first regular use."	Range, 18-24	College freshmen through seniors^c^	Harvard School of Public Health College Alcohol Study, 2001 (n = 10,904)
Sepe et al (29)	Not defined.	Not available.	Not available.	Study-specific search of tobacco industry document archives (sample size not indicated) a
Farrelly et al (30)	"How old were you when you smoked your first cigarette?"	Range, 15-24	Range, 12-24	National Longitudinal Survey of Youth 1997 (n = 8,904)
Richardson et al (30)	Not defined.	Range, 18-24	Range, 12-24	Legacy Media Tracking Survey, December 1999 to January 2004 (n = 19,701)
Hersey et al (32)	Used a definition of smoking progression instead of initiation.^b^	Range, 18-24	Range, 12-24	Legacy Media Tracking Survey I, Fall 1999 and Winter 2000 (n = 6,352)
Wechsler et al (33)	"Age when first started smoking cigarettes regularly."	College freshmen through seniors^c^	College freshmen through seniors^c^	1999 Harvard School of Public Health College Alcohol Study (n = 4,495)
Zhang et al (34)	Did not smoke at baseline (1994-1995) but smoked (daily or occasionally) at follow-up (1996-1997).	Range, 20-24	Range, baseline only, 20-24	Statistics Canada's National Population Health Survey Cycle 1 (1994-1995) and Cycle 2 (1996-1997) (n = 636)

a Smoking progression was defined as moving from 1) *never smoker* (never smoked a cigarette), to 2) *experimenter* (smoked at least 1 but fewer than 100 cigarettes in his or her lifetime but did not smoke in the last 30 days), to 3) *current non-established smoker* (smoked fewer than 100 cigarettes in his or her lifetime and smoked in the last 30 days), to 4) *current established smoker* (smoked 100 cigarettes in his or her lifetime and smoked in the last 30 days).

b Smoking progression was defined as moving from 1) *closed to smoking* (those who had not smoked cigarettes and did not intend to do so), 2) *open to smoking* (respondents who had not smoked cigarettes but indicated that they might smoke in the future), 3) *prior experimenters* (those who had tried cigarettes but who had not smoked during the past month), 4) *early smokers* (respondents who had smoked cigarettes at least once in the past 30 days but were not yet established smokers), and 5) *established smokers* (those who had smoked cigarettes on 20 of the past 30 days and who had smoked 100 or more cigarettes in their lifetime).

c Not clearly identified or defined in study.

**Table 2:** Measures of Smoking Initiation Defined in a Systematic Review of Studies on Smoking Initiation Among Young Adults in the United States and Canada, 1998-2010

**Category and Measure of Smoking Initiation**	Number of Studies Using Measure
**Initiation of smoking**	
"Age at which smoked first whole cigarette."	1
"Age when smoked first whole cigarette."	1
"Age when first tried cigarette smoking."	1
"Age at first use."^a^	1
"Age at which smoked first cigarette."	1
"Ever smoked even a puff of a cigarette in the year after high school."	1
"Age when first smoked a cigarette [more than a puff]."	1
"How old were you when you smoked your first cigarette?"	1
Never-smokers at baseline, but began smoking cigarettes during study.	1
Did not smoke at baseline, but smoked (daily or occasionally) at follow-up.	1
**Initiation of regular smoking**	
"Age when first started smoking cigarettes regularly."	2
"Age when first started to smoke fairly regularly."	1
"Age at first regular use."^a^	1
"Reported regular cigarette smoking (at least 5 cigarettes per week almost every week for at least 3 months)."	1
**Prevalence of smoking among youth**	
"Smoked <100 cigarettes but smoked during the past 30 days."^a^	1
**Prevalence of smoking among adults**	
"Have you smoked at least 100 cigarettes in your entire life?" and "How old were you when you first started smoking cigarettes fairly regularly?"	2
"Smoked at least 100 cigarettes in lifetime."	1
"Lifetime use ≥100 cigarettes."	1
"Smoked ≥100 cigarettes and smoked during the past 30 days."^a^	1
**Smoking progression**	
Used a definition of smoking progression instead of initiation.^b^	2
**Study-specific**	
"Started smoking after joining the military."	2
"Smoking, even a puff, over the past 7 days."	1
**Not clearly defined**	
Not clearly defined other than "age of smoking onset."	1
**Undefined**	
Authors did not define initiation (Richardson et al [31], Sepe et al [29])	2

a Two studies used 2 definitions of initiation; thus the total number of studies in this table add to 29, not 27, the number of studies included in this review.

b Each study used its own definition of smoking progression instead of smoking initiation. See Methods section for exact definitions.

**Table 3:** Studies Addressing Research Question on Which Young Adults Initiate Smoking, Systematic Review of Studies on Smoking Initiation Among Young Adults in the United States and Canada, 1998-2010

**Study**	**Primary Result**
**Sex**	
Lantz (2)	Comparing the 1970 birth cohort with the 1977 birth cohort, the proportion of males who reported becoming regular smokers as young adults increased by 75% (14% vs 25%). Comparing the same cohorts, the proportion of females who reported becoming smokers as young adults increased by only 6% (18% vs 19%).
Reed et al (10)	Among college students who were never smokers 12 months before participation in the survey, males were nearly 1.8 times as likely to start smoking as were females.
Myers et al (11)	Among Chinese and Korean American college students who were never-smokers at baseline (start of freshmen year in college), men were 2.25 times as likely as women to initiate smoking while in college.
Bray et al (12)	Across all service divisions, young adult males had higher rates of smoking initiation than young adult females (21% vs 17%).
**Race/ethnicity**	
Kiefe et al (13)	Comparing initiation rates in 10 years of data among adults who were never-smokers at baseline, African Americans were more than twice as likely as whites to have started regular smoking (7.1% vs 3.5% for females; 13.2% vs 5.1% for males).
Biener and Albers (9)	42% of nonwhite young adult smokers reported initiation of regular smoking after age 18, compared to 27% of young white adult smokers.
Trinidad et al (14)	65.4% of Asian/Pacific Islanders and 52.7% of African American smokers reported initiating smoking between ages 18 and 25.
Viola (15)	Black smokers were 2.5 times as likely to initiate smoking as young adults than as youth. Other racial/ethnic minority smokers were 1.6 times as likely to initiate smoking as young adults than as youth.
Hailpern and Viola (16)	7 years of data (1997-2003) suggest a significant upward trend in the proportion of black males who reported smoking initiation as young adults (16% in 1997 vs 24% in 2003).
Trinidad et al (17)	Greater percentages of African Americans reported initiating regular smoking as young adults (ages 18, 19, and 20) compared to non-Hispanic whites (26% vs 22% at age 18; 17% vs 11% at age 19; 14% vs 10% at age 20).
Watson et al (18)	Among a sample of young adult women, white current-smokers started smoking significantly younger than black current smokers (age 16 vs age 20).
Choi et al (19)	Among never smokers in high school, whites were 1.5 times as likely as nonwhites to progress in their smoking status while in college.
Reed et al (20)	Among college students who reported not smoking 12 months prior to participating in the study, a greater percentage of whites than nonwhites reported initiating smoking while in college (11% vs 6%).
Green et al (21)	Among Air Force technical training students, white nonsmokers were 1.6 times as likely as other nonsmokers to initiate smoking after basic training.
**Education**	
Stockdale et al (22)	Among college students, 13% to 25% of current smokers began smoking at age 18 or older. (Results varied by the time of the survey and by population of students [eg, psychology students] surveyed.)
Choi et al (19)	Of students who were never smokers in high school, 29% reported experimenting with smoking in college, 4% reported progressing to a current nonestablished smoker, and an additional 4% reported progressing to current established smoker while in college.
Staten et al (23)	Undergraduate students who reported smoking their first whole cigarette as young adults were more likely to be juniors or seniors (78%) than freshmen or sophomores (22%). (Although the study reported age as a significant predictor of cigarette initiation, it did not provide specific age ranges associated with year in school.) Among undergraduate students, for every additional year increase in age, the likelihood of smoking initiation while in college increased 35%.
Hailpern and Viola (16)	7 years of data (1997-2003) suggest a significant upward trend in individuals with a college education who report smoking initiation as young adults. Results showed proportions increasing from 11% in 1997 to 14% in 2003. The upward trend occurred across all groups but was most pronounced for college-educated whites (11% to 15%) and for college-educated nonblack racial/ethnic minorities (11% to 13%).
**Age**	
Tercyak et al (24)	Of 12th graders who had never smoked prior to completing high school, 25% tried their first cigarette in the following year.
Biener and Albers (9)	31% of young adult smokers (aged 18 to 30) and 41% of older adult smokers (aged 31 to 65) reported initiating regular smoking after they turned 18.
**Family characteristics**	
Staten et al (23)	Compared to college students whose parents did not attend college, college students with a college-educated parent were 4.8 times as likely to initiate smoking while in college.
Myers et al (11)	Among Chinese and Korean American college students who were never-smokers at baseline (start of freshmen year in college), students with parents who smoked were 2.25 as likely to smoke their first cigarette while in college.
**Previous tobacco use**	
Choi et al (19)	Among college students who experimented with smoking in high school, 25% progressed in their smoking behavior while attending college. Of college-bound high school students who reported smoking in the past 30 days but who reported smoking fewer than 100 cigarettes in their lifetime, 50% progressed to current established smoker while in college.
Haddock et al (25)	Male Air Force recruits who reported current or past smokeless tobacco use were 2.3 times as likely to initiate smoking as recruits who reported never using smokeless tobacco.
**Use of alcohol**	
Tercyak et al (24)	Never smokers in the 12th grade who reported drinking alcohol while in high school (drinking alcohol in the last 30 days) were 1.8 times as likely as nondrinkers to start smoking in the year following graduation from high school.
Reed et al (20)	College students who reported drinking alcohol on 3 to 5 occasions during the past year were 4.5 times as likely to report smoking their first cigarette in the past year, compared to college students who abstained from using alcohol. College students who reported drinking alcohol on 10 or more occasions during the last year were more than 9 times as likely to report smoking their first cigarette in the past year, compared to college students who abstained from using alcohol.
Myers et al (11)	Among Chinese and Korean American college students who were never-smokers at baseline (start of freshmen year in college), those who reported any drinking in the 30 days prior to baseline assessment were 3.65 times as likely to smoke their first cigarette during college than those who reported no drinking during the same period.
Staten et al (23)	College undergraduates who drank alcohol (ie, had at least 1 drink in the last 30 days) were 8.6 times as likely to try smoking as students who did not drink.
Costa et al (26)	Compared to never-smokers who did not initiate smoking during the 3-year study period, students who did initiate smoking had a higher average of instances of problem drinking (eg, greater frequency of drunkenness, experiencing negative consequences of drinking such as problems completing schoolwork).
Viola (15)	Compared to nonsmokers, young adults who smoked at least 100 cigarettes in their lifetime were nearly 3.9 times as likely to consume "moderate-to-large" amounts of alcohol. (Study did not clearly define the amount of alcohol equivalent to "moderate-to-large.")
Reed et al (10)	College undergraduates who reported drinking on 3 or more occasions in the past year were 6.0 times as likely to initiate smoking as college undergraduates who did not drink in the past year. Compared to college undergraduates who did not drink in the past year, college undergraduates who reported drinking on 40 or more occasions were 15.8 times more likely to initiate smoking.
**Use of other illegal drugs**	
Reed et al (10)	College undergraduates who reported using marijuana in the past year were 3.6 times as likely to initiate smoking as students who did not report using marijuana in the last year. College undergraduates who reported using illegal drugs in the past year were 3.5 times as likely to initiate smoking as students who did not report using illegal drugs in the last year. College undergraduates who reported using prescription drugs in the past year without a prescription were 2.3 times as likely to initiate smoking as students who did not report using prescription drugs in the past year without a prescription.
Reed et al (20)	College students who reported using marijuana in the past year were more than 3 times as likely to report smoking their first cigarette in the past year as nonmarijuana users.
Costa et al (26)	Compared to never-smokers who did not initiate smoking during the 3-year study period, those that did initiate smoking had a higher average, as a group, of reported marijuana (or hash) use in the past month.
Myers et al (11)	Among Chinese and Korean American college students who were never-smokers at baseline (start of freshmen year in college), those who reported any use of illegal drugs in the 30 days prior to baseline assessment were 5 times as likely to experiment with smoking for the first time while in college.

**Table 4:** Studies Addressing Research Question on Circumstances Under Which Initiation Among Young Adults Takes Place, Systematic Review of Studies on Smoking Initiation Among Young Adults in the United States and Canada, 1998-2010

**Study**	**Primary Result**
**Exposure to smokers**	
Ling et al (27)	Exposure to smokers (eg, family members, friends, coworkers, and other social contacts who smoke) doubled the susceptibility of never smokers and experimenters (ie, those who smoked fewer than 100 cigarettes) to future smoking.^a^
Green et al (21)	Among trainees who did not smoke before basic training, those who lived with a roommate who smoked were 1.7 times as likely to initiate cigarette use as those who lived with a roommate who did not smoke.
**Exposure to tobacco marketing and social events sponsored by the tobacco industry**	
Rigotti et al (28)	Compared to college students who did not attend tobacco promotional events, exposure to a tobacco promotional event increased the odds of becoming a current smoker 1.7 times among college students who had not smoked regularly before age 19.
**Peer influence and social networks**	
Sepe et al (29)	Tobacco industry documents showed that peer influence was a major factor in promoting smoking initiation among young adults. (Peer influence was not defined by the authors.)
Stockdale et al (22)	College students who reported trying or increasing their smoking since coming to college had significantly more prosmoking social influences (ie, riding in a car with other smokers, allowing someone to smoke in one's home, and having friends who smoke) than students who maintained, decreased, or quit smoking.
Costa et al (26)	Compared to never-smokers who did not initiate smoking during the 3-year study period, students who did initiate smoking reported greater vulnerability to peer pressure to smoke and drink. College students who initiated smoking after their first semester also reported having more friends or acquaintances that engaged in substance use such as marijuana or heavy drinking, compared to college students who never smoked.
Staten et al (23)	Among college students who report initiating smoking, 27% belonged to a fraternity or sorority; among never-initiators, 16% belonged to a fraternity or sorority. Compared to college students who did not participate in service organizations, college students who participated in service organizations were one-fourth as likely (odds ratio = 0.29) to initiate smoking while in college.
**Attitudes and perceptions**	
Stockdale et al (22)	College students who reported trying or increasing their smoking since coming to college had significantly lower antismoking attitudes (ie, having "relationships with smokers," not supporting smoking restrictions in public places, and not supporting college antitobacco policies) than did those who maintained, decreased, or quit smoking.
Choi et al (19)	Among college students who experimented with smoking in high school, those who believed their best friends would approve of their smoking 1 or more packs of cigarettes a day were 2.0 times as likely to progress in their smoking behavior, compared to those who believed their best friends would disapprove of their smoking 1 or more packs of cigarettes a day. Also among college students who experimented with smoking in high school, those who believed experimentation with smoking was either "moderately safe" or "safe" were 1.7 and 1.9 times, respectively, as likely to progress in their smoking behavior than were students who believed smoking was "not safe."
Costa et al (26)	College students who initiated smoking after their first semester reported being less responsive to "social regulation" (eg, having parents or friends who did not disapprove of problem behavior like underage drinking or using marijuana, having friends who would prevent transgressions like academic dishonesty), compared to college students who never smoked. Similarly, college students who initiated smoking after their first semester reported being less concerned with "personal regulation" (eg, personal health was less important, the consequences of health-compromising behavior were downplayed, personal achievement was less important), compared to college students who never smoked.
Green et al (21)	Among trainees who did not smoke before basic training, those who reported that some of their military training leaders and/or classroom instructors used tobacco were 1.7 times as likely to initiate smoking after basic training as trainees who did not report that their training leaders and instructors used tobacco. Among trainees who did not smoke before basic training, those who perceived that most (more than 50%) of their classmates smoked during technical training were 1.7 times as likely to initiate smoking compared with those who perceived that 50% or fewer of their classmates smoked during training.
Bray et al (12)	When asked why they started smoking regularly after joining the military, the top 3 responses given by military personnel across all service divisions were 1) "to help me relax or calm down," 2) "to help relieve stress," and 3) "to relieve boredom."

a This study defined susceptibility to future smoking as never smokers and experimenters (those who reported smoking fewer than 100 cigarettes in their lifetime) who answered anything but "definitely not" to "Do you think you will smoke a cigarette in the next year?"

**Table 5:** Studies Addressing Research Question on Programming and Policy Efforts That Reduce Young Adult Smoking Initiation, Systematic Review of Studies on Smoking Initiation Among Young Adults in the United States and Canada, 1998-2010

**Study**	**Primary Result**
**Counter-industry marketing and denormalization campaign strategies**	
Farrelly et al (30)	A longitudinal cohort study from 1997 to 2004 of adolescents aged 12 to 17 years at baseline found exposure to the Truth campaign may have been successful in preventing as many as 450,000 adolescents and young adults from initiating smoking between 2000 and 2004.
Richardson et al (31)	Results from 8 cross-sectional nationally representative telephone surveys of young adults aged 18 to 24 years between December 1999 and January 2004 found that awareness of the national Truth campaign was significantly associated with greater antismoking attitudes among young adults.
Hersey et al (32)	Compared to young adults in states without counter-industry media campaigns, young adults living in states with aggressive counter-industry media campaigns were more likely to have negative beliefs about tobacco industry practices, including: "Cigarette companies (CCs) lie" (odds ratio [OR] = 1.6); "CCs try to cover up all the bad things they've done" (OR = 1.6); "CCs target teens to replace smokers who die" (OR = 1.6); "CCs deny that cigarettes are addictive" (OR = 2.0); and "CCs deny cigarettes cause cancer and other harmful diseases" (OR = 1.7). Negative beliefs about CCs were strongly associated with negative industry attitudes, which in turn were associated with earlier stages of smoking progression.^a^
**Smoke-free policies**	
Wechsler et al (33)	Among students who were not regular smokers before age 19, current cigarette use was significantly lower for those who lived in smoke-free housing than for those that lived in housing that allowed smoking (10% vs 16.9%).
**Taxation**	
Zhang et al (34)	A longitudinal study of young adults found cigarette price change was significantly associated with smoking initiation. The greater the price reduction, the greater the likelihood of initiating smoking (OR = 1.15), after controlling for individual characteristics, tobacco-control policies, and unobserved variations between Canadian provinces.

a Smoking progression defined as moving from 1) *closed to smoking* (those who had not smoked cigarettes and did not intend to do so), 2) *open to smoking* (respondents who had not smoked cigarettes but indicated that they might smoke in the future), 3) *prior experimenters* (those who had tried cigarettes but who had not smoked during the past month), 4) *early smokers* (respondents who had smoked cigarettes at least once in the past 30 days but were not yet *established smokers*), and 5) *established smokers* (those who had smoked cigarettes on 20 of the past 30 days and who had smoked 100 or more cigarettes in their lifetime).
